# Defective enamel and bone development in sodium-dependent citrate transporter (NaCT) *Slc13a5* deficient mice

**DOI:** 10.1371/journal.pone.0175465

**Published:** 2017-04-13

**Authors:** Armando R. Irizarry, Guirui Yan, Qingqiang Zeng, Jonathan Lucchesi, Matthew J. Hamang, Yanfei L. Ma, James Xiaojun Rong

**Affiliations:** 1 Department of Pathology, Lilly Research Laboratories, Eli Lilly & Company, Indianapolis, IN, United States of America; 2 Lilly China R&D Center, Eli Lilly & Company, Building 8, No. 338, Zhangjiang Hi-Tech Park, Shanghai, People’s Republic of China; 3 Musculoskeletal Research, Lilly Research Laboratories, Eli Lilly & Company, Indianapolis, IN, United States of America; Forsyth Institute, UNITED STATES

## Abstract

There has been growing recognition of the essential roles of citrate in biomechanical properties of mineralized tissues, including teeth and bone. However, the sources of citrate in these tissues have not been well defined, and the contribution of citrate to the regulation of odontogenesis and osteogenesis has not been examined. Here, tooth and bone phenotypes were examined in sodium-dependent citrate transporter (NaCT) *Slc13a5* deficient C57BL/6 mice at 13 and 32 weeks of age. *Slc13a5* deficiency led to defective tooth development, characterized by absence of mature enamel, formation of aberrant enamel matrix, and dysplasia and hyperplasia of the enamel organ epithelium that progressed with age. These abnormalities were associated with fragile teeth with a possible predisposition to tooth abscesses. The lack of mature enamel was consistent with amelogenesis imperfecta. Furthermore, *Slc13a5* deficiency led to decreased bone mineral density and impaired bone formation in 13-week-old mice but not in older mice. The findings revealed the potentially important role of citrate and *Slc13a5* in the development and function of teeth and bone.

## Introduction

Bone and teeth are highly mineralized organs [1, 2]. Calcium phosphate, in the form of nano-sized apatite, is the major mineral component in bone, and in dentin and enamel [3]. In bone and teeth, citrate concentration is 100–400 fold higher than that in plasma and most soft tissues [4]. About 70% of whole body citrate is in bone [5], accounting for 5.5% by weight of the organic matter, and about one sixth of the available apatite surface area [6]. For a long time citrate was thought to regulate demineralization as a calcium-solubilizing agent [7]. However, biophysical studies demonstrated that during apatite crystallization in aqueous solutions, citrate not only stabilized existing nanocrystals but also was responsible for crystal nucleation [8, 9]. Furthermore, citrate was found to be strongly bound to the apatite nanocrystals [6, 10]. This is conserved in fish, avian, and mammalian bones [6, 10], indicating the critical role of citrate in bone across species. Although the role of citrate in the formation of dentin and enamel has not been extensively studied, citrate may also be important given its high abundance in teeth [4]. The outstanding role of citrate in bone and teeth draw intriguing questions on what the sources of citrate are, how citrate abundance is regulated, and whether perturbed citrate homeostasis leads to disorders in the development of teeth and bone.

Osteoblasts, odontoblasts and ameloblasts are cells) responsible for bone, dentin, and enamel formation, respectively. Osteoblasts are responsible for *de novo* synthesis and production of citrate, and provide the citrate that is incorporated into the apatite nanocrystals during bone formation [11]. Conceivably, an alternative source of citrate in the tissues is plasma, where citrate concentration is 50 ~200 μM [11, 12]. Osteoblasts, odontoblasts and ameloblasts may take up citrate through transporters in the plasma membrane and use it for the synthesis of bone and teeth. However, there are no data that conclusively indicate that citrate transporters regulate citrate content in these cell types, and the role of the transporters in osteogenesis and odontogenesis is not completely understood.

Three transporters, namely NaDC1 (SLC13A2), NaDC3 (SLC13A3), NaCT (SLC13A5) have been reported to be expressed in kidney, liver, and other organs responsible for uptake of carboxylate metabolites, including citrate, from plasma [13–15]. Notably among them, NaCT is the only known membrane carrier that preferentially transports citrate over other carboxylates from the circulation [16–18]. NaCT mRNA is highly expressed in human liver and widely detected in rodent liver, brain, testis, kidney and skeletal muscle [15, 16, 18, 19]. Recent studies with microarray analysis also demonstrated NaCT mRNA was expressed in bone [20, 21], and its expression level was upregulated during mouse bone matrix formation [20] and molar tooth development [22]. Deletion of mouse *Slc13a5* had decreased overall growth and shorter body length, indicative of a smaller skeletal size, although the bone or tooth phenotypes were not studied [19].

The role of NaCT in the formation of mineralized organs was implicated in human genetics studies, showing that *SLC13A5* mutations with loss of function were associated with tooth hypoplasia, hypodontia, and gingival hyperplasia [23–25]. A recent study further demonstrated the association of *SLC13A5* mutations with Kohlschütter–Tönz syndrome, characterized by epileptic encephalopathy, intellectual disability and amelogenesis imperfecta (AI), hereditary disorders with developmental abnormalities in the quantity and quality of enamel [26, 27]. In the current study, abnormalities in teeth and bone of *Slc13a5* deficient (*Slc13a5*^*-/-*^) mice were characterized. The findings indicate that NaCT may have an important role in the formation of bone and teeth, and *Slc13a5*^*-/-*^ mice potentially represent a novel nonclinical model of AI in human.

## Materials and methods

### Experimental design and animals

*Slc13a5*^*-/-*^ C57BL/6 mice were generated in Taconic (Köln, Germany) and bred in HD Biosciences Co (HDB, Shanghai, China) (S1 Fig and S1 Text). *Slc13a5*^*-/-*^ mice were born alive with no noticeable external abnormality. The male/female ratio and litter size were normal. The mice were not evaluated for neurological disorders in detail because there were no clinical signs of behavioral abnormalities, seizure, or tremor. Evaluation of teeth and bones was conducted using 13 and 32-week-old female littermates including *Slc13a5*^*-/-*^ (homozygotes), *Slc13a5*^*+/-*^ (heterozygotes) and *Slc13a5*^*+/+*^ (wide type, WT) mice. All mice in this study were maintained on a 12-hour light/12-hour dark cycle at 22°C with ad libitum access to chow food (Keaoxieli, Beijing, China) and water, 5~6 mice/cage. All animal procedures and experiments were performed according to protocols approved by the IACUC of Taconic and HDB.

### Calcein incorporation into bone and tissue collection

The activity of bone formation was determined by incorporation of calcein into bone. Calcein is spontaneously fluorescent, and binds to calcium phosphate deposited into the bone matrix only in the actively forming bone or newly synthesized bone sites. Mice were injected subcutaneously with 0.5% calcein solution (Sigma Aldrich, St. Louis, MO, USA, 100 μL/mice) 10, 9, 3, and 2 days prior to euthanasia (one injection each day, a total of four injections each mice). On the day of necropsy, mice were euthanized via CO_2_ inhalation and blood was collected via cardiac puncture into clotting tubes (Gene Era Biotech, Hangzhou, China). The skin and internal organs were removed, and the whole carcass, including the teeth and skeleton, was immersion-fixed in 10% formalin for 24 hours, and then transferred and stored in 70% ethanol until further analysis.

The calcein content in femora was extracted and measured by fluorescence intensity. This quantitative measurement determines the total calcein incorporated into the entire bone segment. Briefly, femora were cut to evaluate distal metaphysis length and subject to demineralization for 1 week in 14% EDTA, pH = 7.2. The EDTA extraction was then diluted 1:1 with water, and an aliquot was removed for measurement of calcein on a Cytoflour II fluorescence plate reader (Thermo Fisher Scientific, Waltham, MA, USA) using excitation and emission wavelengths of 485 and 530 nm, respectively, as previously described [28].

### Serum biochemical assays

The serum level of intact osteocalcin (OCN, Alfa Aesar, Ward Hill, MA, USA) and type I of C-terminal telopeptide collagen (CTX-I, ImmunodiagnosticSystems Ltd, Maryland, MD, USA) were measured using commercially available kits according to manufacturers’ instructions. Serum concentrations of calcium and inorganic phosphorous were measured with a Hitachi Modular analytics automated chemistry analyzer (Roche Diagnostics, Indianapolis, IN, USA).

### Histological evaluation of teeth

After removal of the skull from the carcasses, the crania and mandibles were separated. The crania and the right hemi-mandible were decalcified, trimmed, embedded in paraffin or plastic, sectioned, and stained with hematoxylin and eosin (HE). Undecalcified portions of the left mandible were embedded in plastic to generate ground cross sections at the level of the first molar, and were subsequently stained with Stevenel’s blue. The tissue sections were evaluated via routine light microscopy by an American College of Veterinary Pathologists (ACVP)-certified pathologist. The severity of a microscopic finding was graded on a progressive 4-point scale consisting of minimal (MI), slight (SL), moderate (MO), and marked (MA), with MI being the mildest possible grade, i.e. a change that was minimally outside of normal, and MA being the greatest possible grade, i.e. a change of very great intensity or extent. “Present, no severity grade assigned” was used only to denote the presence of abscesses. WT mice were used as the reference when microscopically evaluating tissues from *Slc13a5*^+/-^ and *Slc13a5*^-/-^ mice.

### Micro-Computed Tomography (μCT)

Femora and 5^th^ lumbar vertebrae (LV5) were analyzed by quantitative μCT using an LTC-100 μCT scanner (Hitachi-Aloka, Tokyo, Japan) with an automatic dose setting and with mouse bone mode at 60 μm resolution. Scans of the femora were taken at 0.4 and 4.4 mm from the end of the growth plate for distal metaphysis and midshaft analyses, respectively. Bone mineral content (BMC) and bone area were reported using Aloka software (SYS-C320 version 1.5) as previously described [29]. Bone mineral density (BMD) was calculated as BMC normalized to bone volumetric area [29]. μCT measurements of left hemi-mandibles were conducted at Charles River Laboratories Montreal (Senneville, Québec, Canada), with a Scanco μCT 100 scanner (Scanco Medical AG, Brüttisellen, Switzerland) with a dose of 70 kVp, 114 μA, 8 W and at a isotropic voxel resolution of 5 μm. The software used for acquisition was μCT v6.1 (Scanco Medical AG, Brüttisellen, Switzerland) and the software used for analyses was μCT Evaluation v6.5–3 (Scanco Medical AG, Brüttisellen, Switzerland). The evaluation was completed prior to processing portions of the left mandible to ground plastic sections (see above). Evaluation included the determination of enamel (incisor and molar) and pulp volume in a gated area in the left mandible (see S2 Fig and S2 Text for details).

### Biomechanical analyses

Biomechanical properties of the mid femur (MF) were evaluated using three-point bending [29]. Specimens were loaded in a 37°C saline bath after being submerged for 2 minutes to allow for equilibration of temperature. The femur length was measured using calipers (Mitutoyo, Kanagawa, Japan), and load was applied midway between two supports 8 mm apart. Femora were positioned so that the loading point was about 4 mm proximal from the distal popliteal space and bending occurred about the medial-lateral axis. Load-displacement curves were recorded at a crosshead speed of 0.17 mm/sec using an MTS model 1/S materials testing machine with a 100N load cell and analyzed using TestWorks 4 software (MTS Corp., Minneapolis, MN, USA).

### Statistical analyses

All numerical data were expressed as mean ± standard deviation (SD). GraphPad Prism 6 was used to analyze differences between samples by 1-way ANOVA with the post-hoc Tukey multiple comparison test. P < 0.05 was considered statistically significant.

## Results

### *Slc13a5*^-/-^ mice were smaller and had an abnormal dental phenotype

**Fig 1:**
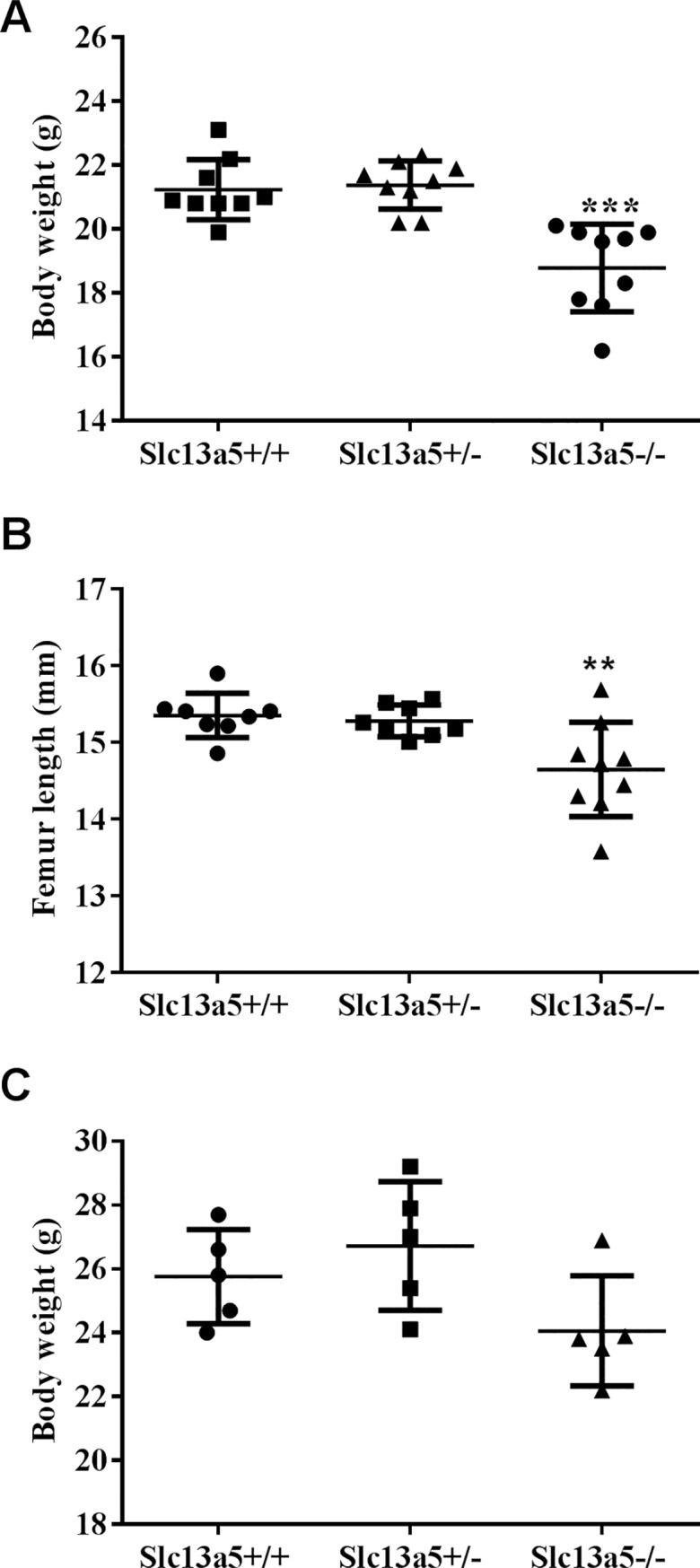
*Slc13a5*^-/-^ mice were smaller in size. (A) Body weight and (B) femur length were less in 13-week-old *Slc13a5*^-/-^ mice (n = 9/group) when compared to WT (*Slc13a5*^*+/+*^) or heterozygous (*Slc13a5*^*+/-*^) littermates. (C) Body weight of 32-week-old mice. ** *P* < 0.01; *** *P* < 0.001 compared with *Slc13a5*^*+/+*^ group.

Thirteen-week-old *Slc13a5*^*-/-*^ mice were smaller in size, with decreased body weight and shorter femur length when compared to *Slc13a5*^*+/-*^ or *Slc13a5*^*+/+*^ littermates (Fig 1A and 1B). At 32 weeks, body weight of *Slc13a5*^*-/-*^ mice was less than that of the WT and the heterozygotes, though not statistically significant (Fig 1C).

**Fig 2:**
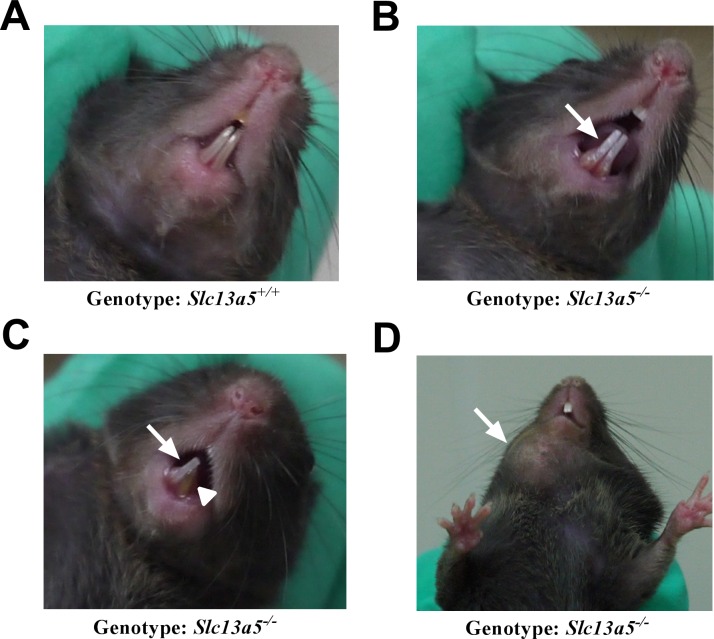
Abnormal dental phenotypes in *Slc13a5*^-/-^ mice. Representative pictures from WT (*Slc13a5*^*+/+*^) (A) and *Slc13a5*^-/-^ (B-D) mice. *Slc13a5*^-/-^ mice had white, opaque areas of discoloration on the incisors (B and C, solid arrows), fractured incisor (C, arrowhead), and/or abscess (D, solid arrow).

Small white, opaque areas of discoloration were observed on the labial surface of the incisors of some of the *Slc13a5*^*-/-*^ mice (Fig 2B), whereas incisors from *Slc13a5*^+/-^ (not shown) or *Slc13a5*^+/+^ (Fig 2A) littermates looked normal. Furthermore, incisors from the *Slc13a5*^*-/-*^ fractured readily (Fig 2C). Some *Slc13a5*^*-/-*^ mice had discrete mandibular swellings which correlated with tooth and mandibular abscesses in 13-week-old mice (Fig 2D and Table 1).

**Table 1:** Microscopic findings in 13-week-old *Slc13a5*^-/-^, *Slc13a5*^+/-^, and *Slc13a5*^+/+^ mice (n = 9).

Tissue	Findings	Group		
		*Slc13a5*^+/+^	*Slc13a5*^+/-^	*Slc13a5*^-/-^
Incisors	Enamel hypoplasia	—	—	9 MA
	Aberrant enamel matrix	—	—	1 MA
				4 MO
				4 SL
	Dysplasia/disorganization, enamel organ epithelium	—	—	4 MA
				2 MO
				3 SL
	Hyperplasia, enamel organ epithelium	—	—	5 MI
				1 SL
Molars	Abscess with bacteria	—	—	2P

— = finding not occur.

MI = minimal; SL = slight; MO = Moderate; MA = Marked; P = Present, no severity grade assigned; the numbers in front of the microscopic findings indicate the number of the mice identified with the findings.

### *Slc13a5*^-/-^ mice lacked mature enamel

**Fig 3:**
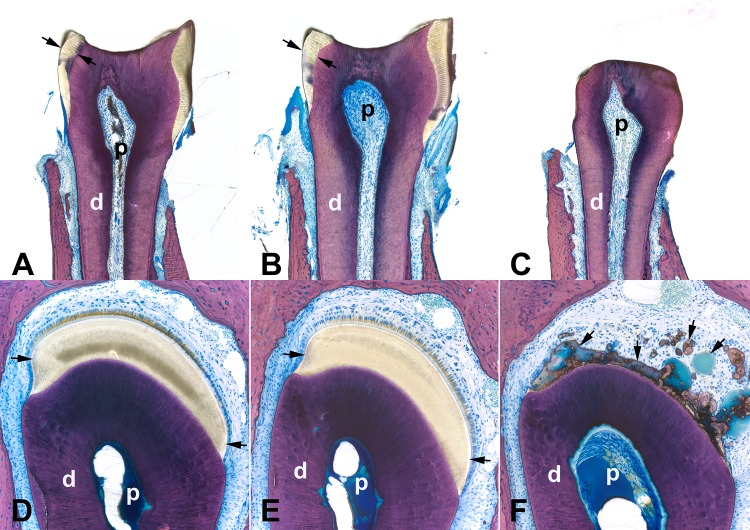
*Slc13a5*^-/-^ mice lacked mature enamel. Representative images of ground thick plastic sections of the first molar (A-C) and incisor (D-F, at the level of the first molar) from the left mandible from 32-week-old *Slc13a5*^+/+^ (A, D), *Slc13a5*^+/-^ (B, E) and *Slc13a5*^-/-^ (C, F) mice. The first molars (C) and incisors (F) of *Slc13a5*^-/-^ mice lacked mature mineralized enamel when compared to *Slc13a5*^+/+^ and *Slc13a5*^+/-^ mice. Unmineralized to poorly mineralized aberrant matrix was present where mature enamel should have been located (F, arrows). Stevenel’s blue. Black arrows in A-B, D-E = mature enamel, black arrows in F = unmineralized (blue green material) or poorly mineralized aberrant enamel matrix. d = dentin; p = pulp.

Because of the macroscopic tooth abnormalities, the dental phenotypes of mice were characterized microscopically. Dentition was complete in mice of all genotypes at 13 and 32 weeks of age. Teeth from *Slc13a5*^+/-^ and *Slc13a5*^+/+^ mice were microscopically normal. However, a complete lack of mature mineralized enamel occurred in incisors and molars of all *Slc13a5*^-/-^ mice at both 13 and 32 weeks of age as evaluated via light microscopy using un-decalcified ground plastic sections (Tables 1 and 2; indicated as enamel hypoplasia; Fig 3). Some 13-week-old mice had tooth and mandibular abscesses (Table 1); however, no abscesses were identified in 32-week-old mice (Table 2).

**Table 2:** Microscopic findings in 32-week-old *Slc13a5*^-/-^, *Slc13a5*^+/-^, *Slc13a5*^+/+^ mice (n = 5).

Tissue	Findings	Group		
		*Slc13a5*^+/+^	*Slc13a5*^+/-^	*Slc13a5*^-/-^
Incisors	Enamel hypoplasia	—	—	5 MA
	Aberrant enamel matrix	—	—	5 MO
	Dysplasia/disorganization, enamel organ epithelium	—	—	3 MA
				2 MO
	Hyperplasia, enamel organ epithelium	—	—	3 MO
				1 MI
				1 SL

— = finding did not occur.

MI = minimal; SL = slight; MO = moderate; MA = marked; the numbers in front of the microscopic findings indicate the number of the mice identified with the findings.

**Fig 4:**
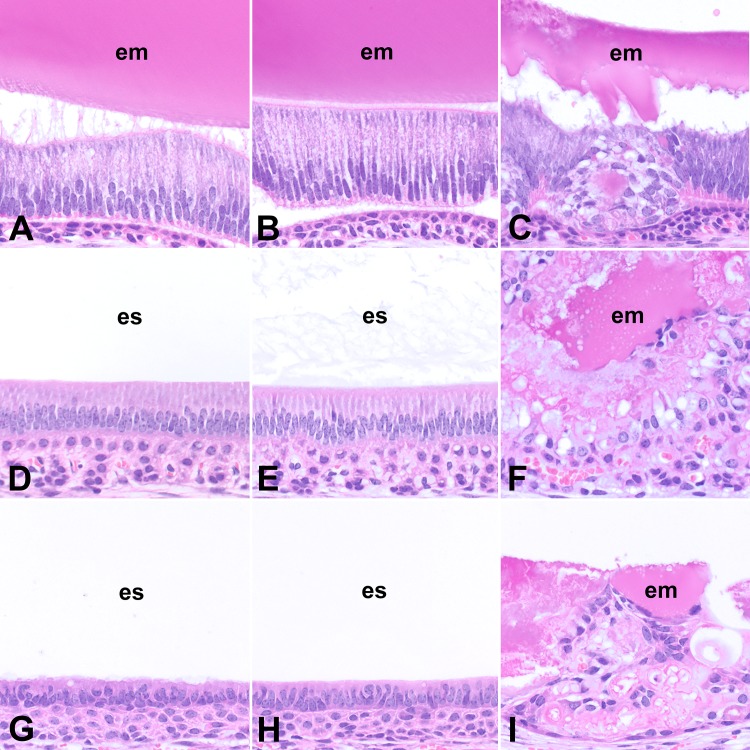
The enamel organ epithelium of the incisors of *Slc13a5*^-/-^ mice was disorganized and dysplastic in all stages of amelogenesis. Representative images of decalcified, paraffin-embedded sections of lower incisors (A-I) from 32- week-old *Slc13a5*^+/+^ (A, D, G), *Slc13a5*^+/-^ (B, E, H) and *Slc13a5*^-/-^ (C, F, I) mice. Images A to C were from the secretory stages of amelogenesis of the incisors near the apex of the tooth, D to F from the maturation stages of amelogenesis at the level of the first molar, and G to I from the post-maturation stages of amelogenesis near the site of eruption. Hematoxylin and Eosin. em = enamel matrix; es = enamel space.

In addition to the lack of mature mineralized enamel, there were other microscopic abnormalities in the incisors of *Slc13a5*^-/-^ mice as evaluated using un-decalcified sections (Fig 3) and decalcified, paraffin-embedded tissue sections (Fig 4). While amelogenesis in the incisors of *Slc13a5*^+/-^ and *Slc13a5*^+/+^ mice was morphologically normal at all stages of amelogenesis (Fig 4), *Slc13a5*^-/-^ mice had abnormal and persistent enamel matrix (indicated as aberrant matrix in Tables 1 and 2), and dysplasia/disorganization and hyperplasia of the enamel organ epithelium (Fig 4, Tables 1 and 2). In the secretory stages of amelogenesis in *Slc13a5*^-/-^ mice, the enamel matrix was lesser in amount when compared to *Slc13a5*^+/-^ and *Slc13a5*^+/+^ mice, and was characterized by irregular aggregates of extracellular eosinophilic matrix of variable staining intensity (Fig 4C), rather than the consistent uniform appearance expected of normal enamel matrix at this stage (Fig 4A and 4B). The enamel organ epithelium of *Slc13a5*^-/-^ mice had multifocal areas of disorganization, characterized by rosette-like epithelial structures surrounding aggregates of eosinophilic extracellular matrix (Fig 4C).

During normal amelogenesis there was progressive degradation of the organic content of maturing enamel, and this appeared in decalcified tissue sections as a clear space (“enamel space”; Fig 4D, 4E, 4G and 4H). When compared to *Slc13a5*^+/-^ and *Slc13a5*^+/+^ mice, the maturation and post-maturation stage of amelogenesis in *Slc13a5*^-/-^ mice were characterized by an enamel space that was reduced in size and retained irregular, globular, often large aggregates of eosinophilic extracellular enamel matrix (Fig 4F and 4I). The aberrant matrix was unmineralized to poorly mineralized, as determined using undecalcified plastic sections (Fig 3F), and did not have the expected organized striated and prismatic structure of normal enamel (i.e. striae of Retzius, enamel prisms). Surrounding the aberrant matrix were rows and ribbons of enamel organ epithelium, which exhibited dysplasia/disorganization and hyperplasia (increased numbers of cells) (Fig 4F and 4I). These changes were present throughout the length of the enamel organ of the incisors, although the most pronounced areas of disorganization and hyperplasia occurred in the maturation and post-maturation stage of amelogenesis. In some of the *Slc13a5*^-/-^ mice the enamel organ epithelium formed a few small cyst-like structures with clear to granular eosinophilic contents (not shown). The microscopic changes were of greater severity in 32-week-old mice when compared to 13-week-old mice, indicating that the changes progressed with age (Tables 1 and 2). *Slc13a5*^*-/-*^ mice had no light microscopic abnormalities in odontoblasts, dentin, or other areas of teeth.

### *Slc13a5*^-/-^ mice had decreased enamel volume

**Fig 5:**
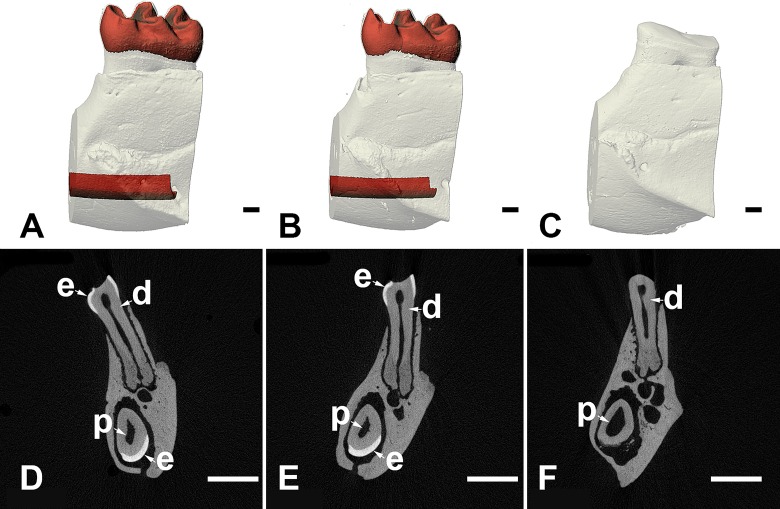
μCT evaluation showed *Slc13a5*^*-/-*^ mice lacked mature, densely-mineralized enamel in the molars and incisors. Representative μCT images of volumetric renderings (A-C) or slices (D-F) of first molars and incisors from 32-week-old WT (A, D), *Slc13a5*^+/-^ (B, E) and *Slc13a5*^-/-^ (C, F) mice. Tissue colored in red in panels A and B corresponds to mature enamel in molars or incisors. Tissue colored white in panels A to C corresponds to bone or dentin, both of which have generally similar radio-density. Enamel (e), dentin (d), and pulp (p) are indicated in molars (top) and incisors (bottom) in panels D through E. Size bar in panels A-C = 100 micrometers; size bar in panels D-E = 1 millimeter.

**Fig 6:**
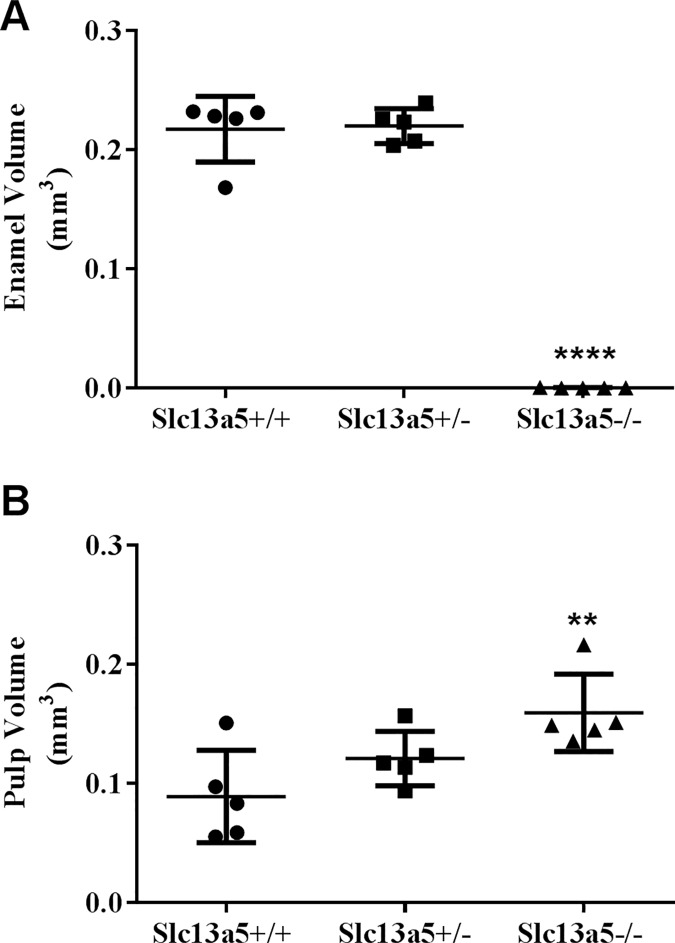
*Slc13a5*^*-/-*^ mice had decreased enamel volume as measured with μCT. (A) 32-week-old *Slc13a5*^-/-^ mice lacked mature enamel as measured by μCT when compared to *Slc13a5*^+/-^ and *Slc13a5*^+/+^. (B) Pulp volume was modestly increased in *Slc13a5*^-/-^ mice. Mice were 32-week-old, n = 4~5/group. ** *P* < 0.01; **** *P* < 0.0001 compared with *Slc13a5*^*+/+*^ group. Volume was measured in mm^3^.

To further characterize the microscopic changes in enamel observed in the *Slc13a5*^*-/-*^ mice, left mandibles of 32-week-old *Slc13a5*^*-/-*^ mice and their heterozygous and WT littermates were analyzed by μCT (Figs 5 and 6). Visual evaluation of the images and morphometric analysis confirmed absence of enamel in *Slc13a5*^*-/-*^ mice (Figs 5C, 5F and 6A). *Slc13a5*^*-/-*^ mice lacked the layer of densely radio-opaque enamel in the incisors and molars (Fig 5). The incisor pulp volume (PUV) (Fig 6B) of *Slc13a5*^*-/-*^ mice was increased when compared to *Slc13a5*^*+/+*^ mice. The cause for the increase in PUV was unclear, and may have been due to decreased thickness of the dentin layer of the incisor (Fig 5F). However, no microscopic differences in the structure or thickness of dentin were identified in tissue sections. Enamel volume and PUV in heterozygous littermates did not differ from that of WT.

### *Slc13a5* deficiency led to decreased bone mineral density and impaired bone formation

**Fig 7:**
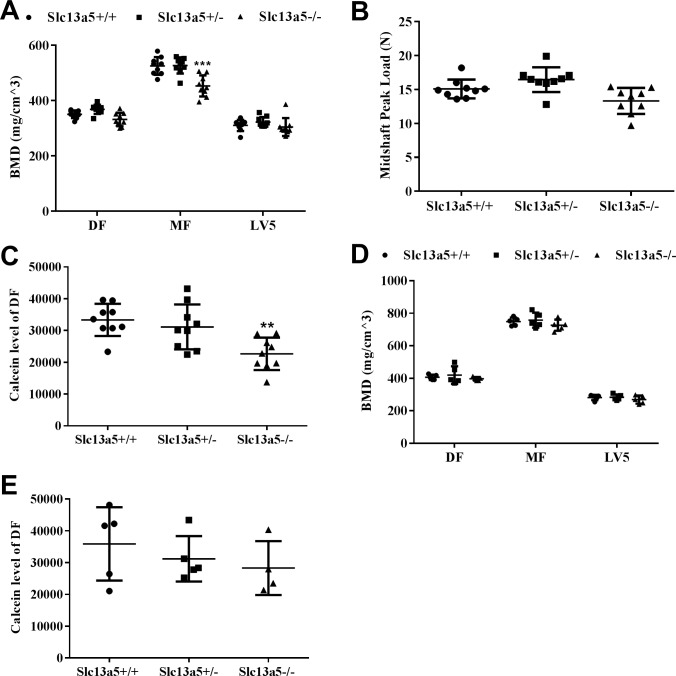
Measurements of bone mineral density, strength and formation. At 13 weeks old, (A) *Slc13a5*^-/-^ mice had similar BMD in distal femur (DF) and 5^th^ lumbar vertebrae (LV5) to *Slc13a5*^*+/+*^ mice, but had decreased BMD in mid femur (MF), and a trend with decreased bone strength (B) (P = 0.096), and decreased calcein incorporation (C) compared with *Slc13a5*^*+/+*^ (n = 9 each group). At 32 weeks old, *Slc13a5*^-/-^ mice had similar BMD in DF, LV5 and MF (D) and calcein incorporation (E) to *Slc13a5*^*+/+*^ (n = 5 each group). ** *P* < 0.01; *** *P* < 0.001 vs age-matched *Slc13a5*^*+/+*^ mice.

Bone and teeth share many common mechanisms in biomineralization during formation and development, and therefore, the effect of *Slc13a5* deficiency on bone was studied. μCT analysis showed that BMD of distal femur (DF) and LV5 was similar between each group, while BMD of mid femur (MF) was significantly decreased by 14% in *Slc13a5*^*-/-*^ mice when compared to *Slc13a5*^*+/+*^ (Fig 7A). Meanwhile, *Slc13a5*^*-/-*^ mice trended (p = 0.096) to have lower midshaft peak load in the bone three-point bending biomechanical analyses (Fig 7B). Furthermore, incorporation of calcein in femur, injected prior to sacrifice to assess dynamic bone formation activity, was significantly decreased, by 32% in *Slc13a5*^*-/-*^ mice (Fig 7C). These results suggested bone mineralization and formation was impaired in *Slc13a5*^*-/-*^ mice. In the 32- week-old mice, BMD of MF, DF, and LV5 were comparable among all groups (Fig 7D). There was no significant difference in calcein incorporation in femur (Fig 7E).

### Serum concentrations of calcium, inorganic phosphorus, OCN, and CTX-I

**Fig 8:**
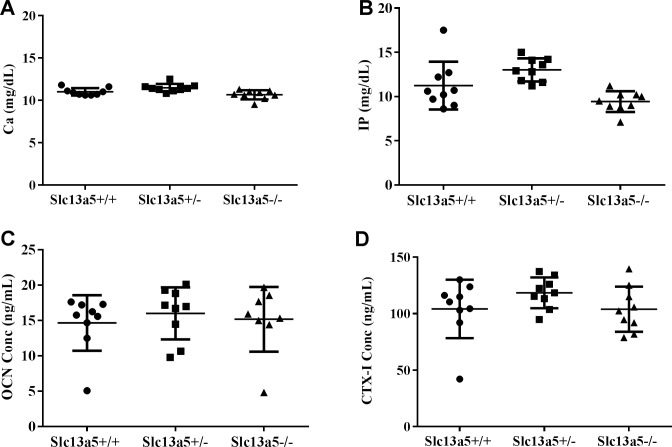
Serum concentrations of calcium, inorganic phosphorus, OCN, and CTX-I. Serum concentrations of calcium (A), inorganic phosphorus (B), intact OCN (C), and CTX-I (D) were similar among 13-week-old *Slc13a5*^*+/+*^, *Slc13a5*^+/-^ and *Slc13a5*^-/-^ mice (n = 9/group).

As calcium and inorganic phosphorus (IP) are the main inorganic component of apatite nanocrystal in teeth and bone, their concentrations in serum were evaluated for potential systemic changes that may have contributed to the dental and skeletal phenotypes in *Slc13a5*^*-/-*^ mice. *Slc13a5*^*-/-*^ mice had similar serum concentrations of calcium and IP when compared to *Slc13a5*^+/-^ and *Slc13a5*^+/+^ at 13 weeks of age (Fig 8A and 8B). There were no changes in serum bone formation marker intact OCN and bone resorption marker CTX-I (Fig 8C and 8D). These results suggested that deficiency of enamel and differences in bone mineral density were not caused by abnormal serum concentrations of calcium and IP, but rather due to a local effect in teeth and bone.

## Discussion

The current study examined the tooth and bone phenotypes in C57BL/6 mice deficient in *Slc13a5*. We have demonstrated and further characterized, in a preclinical model, that *Slc13a5* deficiency results in defective tooth development with morphologic characteristics similar to hypoplastic AI described in human [26]. Collectively, the dental phenotypes of *Slc13a5*^-/-^ and the reported association of tooth and enamel abnormalities in patients with *SLC13A5* loss of function mutations [23–26] indicate that NaCT plays a critical role in tooth development. Furthermore, *Slc13a5* deficiency led to decreased bone mineral density and impaired bone formation in young adult mice, revealing the potentially important role of NaCT and citrate in the formation and function of bone.

Citrate is highly enriched in bone and other mineralized organs [4, 5], and has recently been demonstrated to be essential for the organization of the bone apatite nanocrystals [6, 10]. Osteoblasts [11], and possibly odontoblasts and ameloblasts, are responsible for synthesis of the citrate incorporated into the apatite nanocrystals in bone and teeth. Citrate in these cells is derived from de novo citrate synthesis, predominantly in mitochondria, and from uptake from plasma. Knockout of the mitochondrial citrate transporter *Slc25a1*, responsible for transport of citrate from mitochondria to cytosol, resulted in notable reductions in the number of osteoblasts and the amount of osteoid in mice [30], underscoring the important role of citrate in osteoblast differentiation and formation of osteoid. An alternative source of cellular citrate is uptake from plasma through membrane carboxylate transporters, including NaCT [12]. The expression level of NaCT mRNA in bone and primary osteoblasts/osteocytes, and teeth, was confirmed in the current study (S3 Fig and S3 Text), consistent with the earlier findings [20–22, 26, 31, 32]. *Slc13a5* deficiency led to defective tooth and bone development in mice from our study, demonstrating the importance of the citrate transporters in the biology of these tissues.

Our findings are supported by several previous reports that evaluated NaCT expression in teeth and bone. NaCT mRNA was upregulated in developing molar teeth from postnatal mice [22]. Furthermore, in human tooth buds, NaCT mRNA expression level increased during differentiation of pre-ameloblasts into secretory ameloblasts [32], potentially linking the transporter to enamel formation. NaCT mRNA was upregulated and its expression level peaked during the synthetic phase of bone formation induced by mechanical loading [20], potentially implicating NaCT in the synthetic activity of osteoblasts. In the Hyp mouse homolog of X-linked hypophosphatemic rickets/osteomalacia, a model with impaired bone mineralization, NaCT mRNA expression was decreased in bone, and this was thought to deprive osteoblasts of citrate and increase local extracellular citrate levels, which may have contributed to impaired mineralization [21].

*Slc13a5* deficiency appeared to have differential effects on the mineralized tissues in our study. In *Slc13a5*^-/-^ mice, mature enamel did not form, and the data suggested that amelogenesis was potentially impaired at the secretory and maturation stage (Fig 4). The enamel matrix that did form was lesser in amount, was unmineralized to poorly mineralized, was not degraded during maturation of enamel as expected in normal mouse incisor, and did not have the organized striated and prismatic microscopic structure of normal mature enamel [33]. Schossig et al. in their case report of AI of the hypoplastic type in patients with mutations in *SLC13A5* [26] indicated that the tooth of one patient had decreased thickness of the enamel layer, and the enamel did not have the organized striated and prismatic microscopic structure of normal mature enamel. *Slc13a5*^-/-^ mice appeared to have a more severe disturbance in enamel than that described by Schossig et al [26] in one patient. Similar to the reported patients [26], there were no microscopically detectable abnormalities in dentin in *Slc13a5*^-/-^ mice, although μCT evaluation of the teeth from *Slc13a5*^-/-^ mice suggested that the layer of dentin was potentially thinner, as evidenced by small increase in the volume of the pulp cavity (Fig 5). The discrepancy between the microscopic and μCT evaluation was likely related to the limitations of the two methods, and the small magnitude of the difference in pulp volume, which suggested that the possible difference in dentin thickness was small. Nevertheless, our data suggested that *Slc13a5*^-/-^ mice potentially represent a novel *in vivo* preclinical model of AI, and highlighted the important role of the transporter in amelogenesis in human and other animals.

Bone mineral density decreased and bone formation was impaired in 13-week-old mice, but there were no differences in BMD and bone formation in 32-week-old mice. The transient effects in the bones of younger mice suggest NaCT plays an important role in bone formation and growth, but NaCT may not be as critical in the maintenance of a mature skeleton. The different effects on enamel, dentin, and bone may be due to differences in the mineralization matrix and the mode of mineralization. Enamel forms by apatite crystallization on a noncollagenous protein matrix, which is secreted from ameloblasts and immediately mineralized. In contrast, bone and dentin form similarly on a preformed, unmineralized, collagenous matrix [1]. The crystals in bone and dentin are of a similar size, and about ~10 times smaller in all dimensions than enamel crystals. During enamel formation, the immediate need for mineralization [1], and the extent to which enamel depends on citrate in crystal nucleation, thickening and stabilization could potentially explain why the tissue is more susceptible than bone and dentin to perturbed citrate homeostasis due to *Slc13a5* deficiency. Furthermore, the lack of citrate uptake may be uniquely detrimental to amelogenesis in *Slc13a5* deficient mice, because citrate may regulate cellular acid-base balance, which is critical during the formation of crystals in amelogenesis, demonstrated when sodium bicarbonate homeostasis and intracellular pH regulation was disrupted in patients with mutations of *SLC4A4* and in *Slc4a4* deficient mice [27].

In conclusion, the roles of NaCT in teeth and bone were studied in *Slc13a5*^-/-^ mice. *Slc13a5* deficiency resulted in abnormalities in the enamel and bone, demonstrating the importance of the transporter in amelogenesis and osteogenesis. While there is pharmaceutical interest in targeting NaCT as a potential treatment for insulin resistance and non-alcoholic steatohepatitis [19, 34, 35], one must take into consideration the potential risks highlighted by human [23–26, 36] and /or rodent data, mainly epilepsy, developmental delays, and bone and tooth disorders.

## Supporting information

S1 Fig (TIF)

S2 Fig (TIFF)

S3 Fig (TIF)

S1 Text (DOCX)

S2 Text (DOCX)

S3 Text (DOCX)
