# Supplementary figures and images for: Defective enamel and bone development in sodium-dependent citrate transporter (NaCT) Slc13a5 deficient mice
Source: PLoS One. 2017 Apr 13;12(4):e0175465. doi: 10.1371/journal.pone.0175465 (PMC5391028; doi:10.1371/journal.pone.0175465)

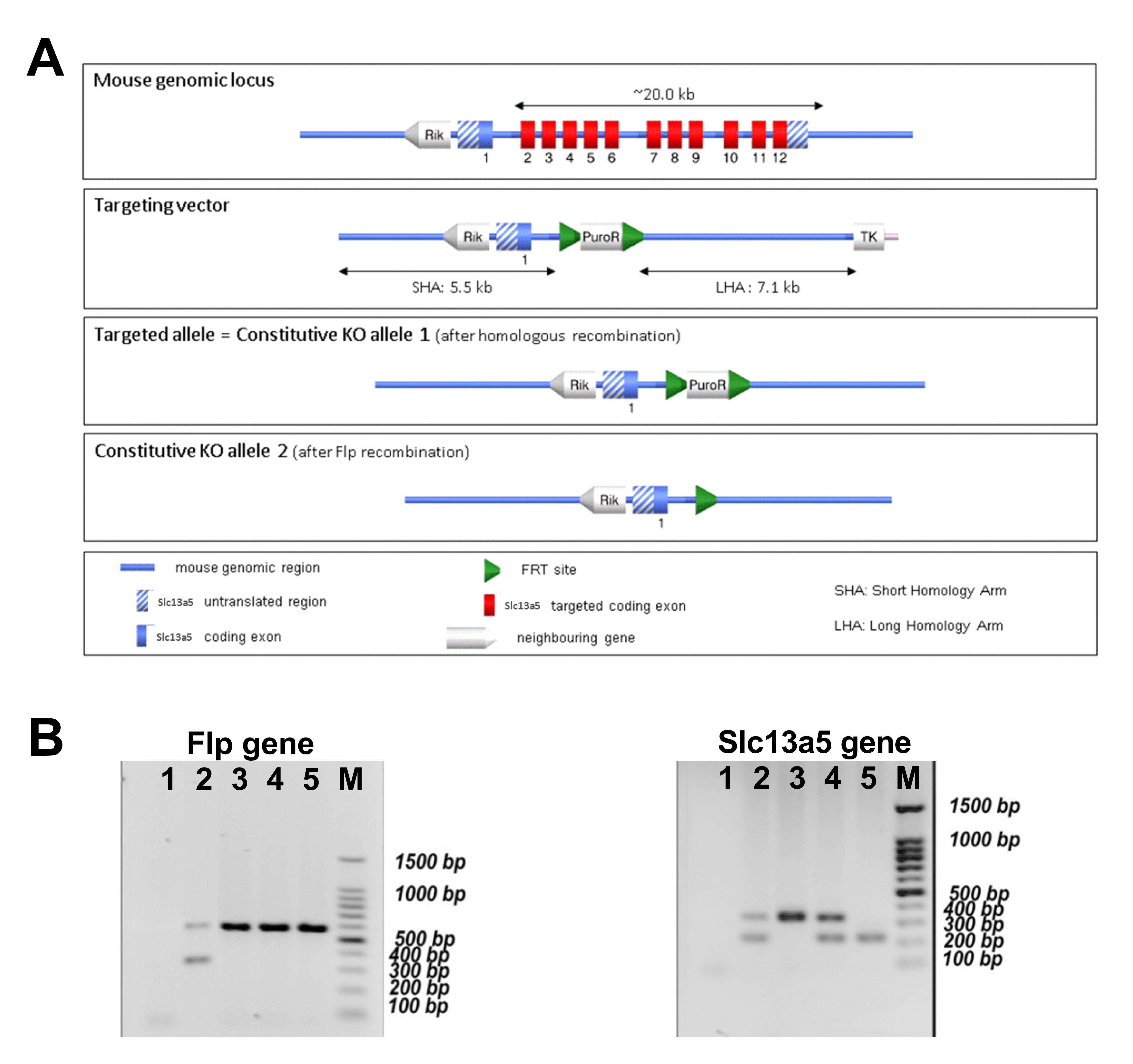

Supplement: S1 Fig — (TIF) [file pone.0175465.s001.tif]

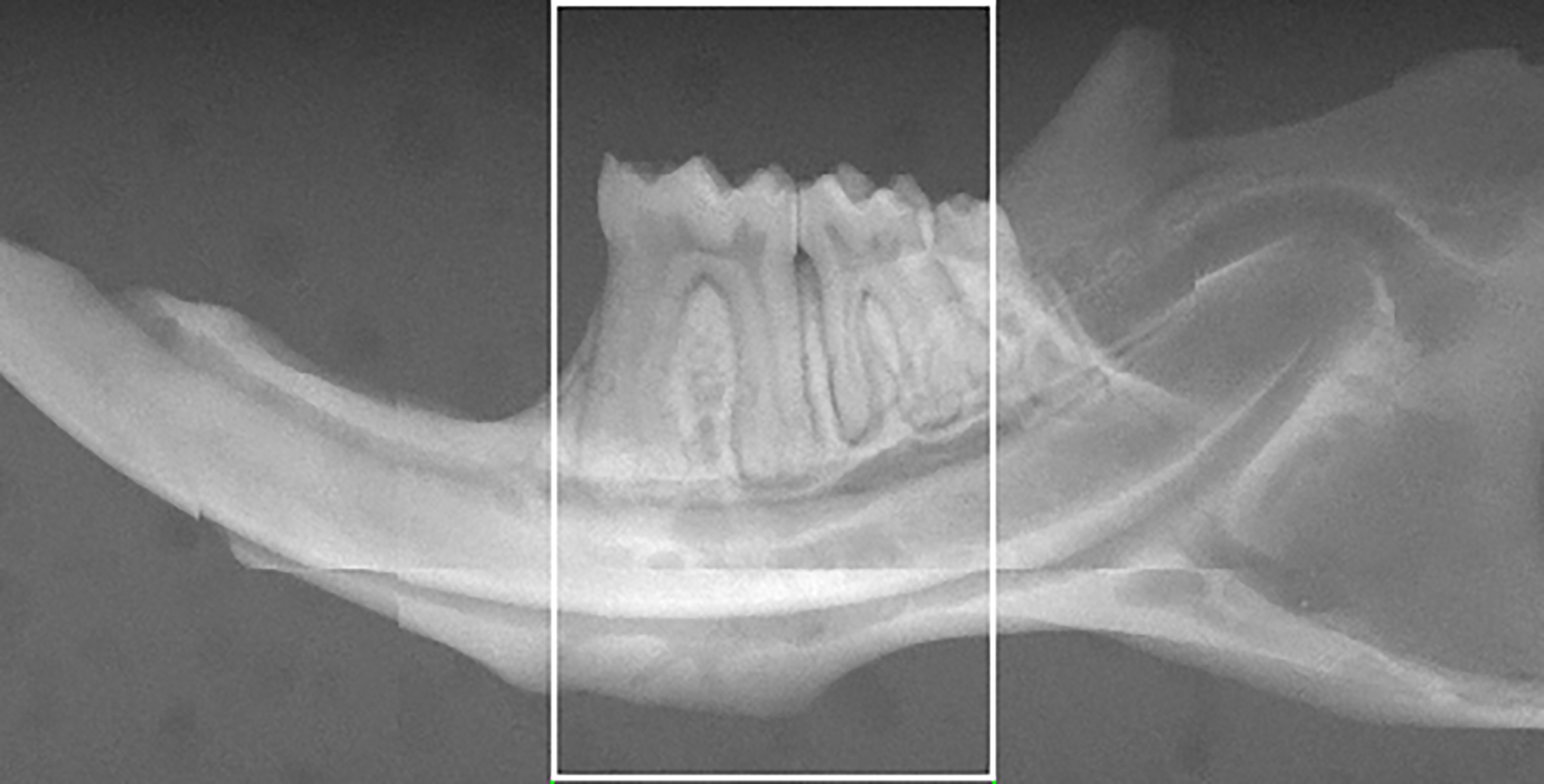

Supplement: S2 Fig — (TIFF) [file pone.0175465.s002.tiff]

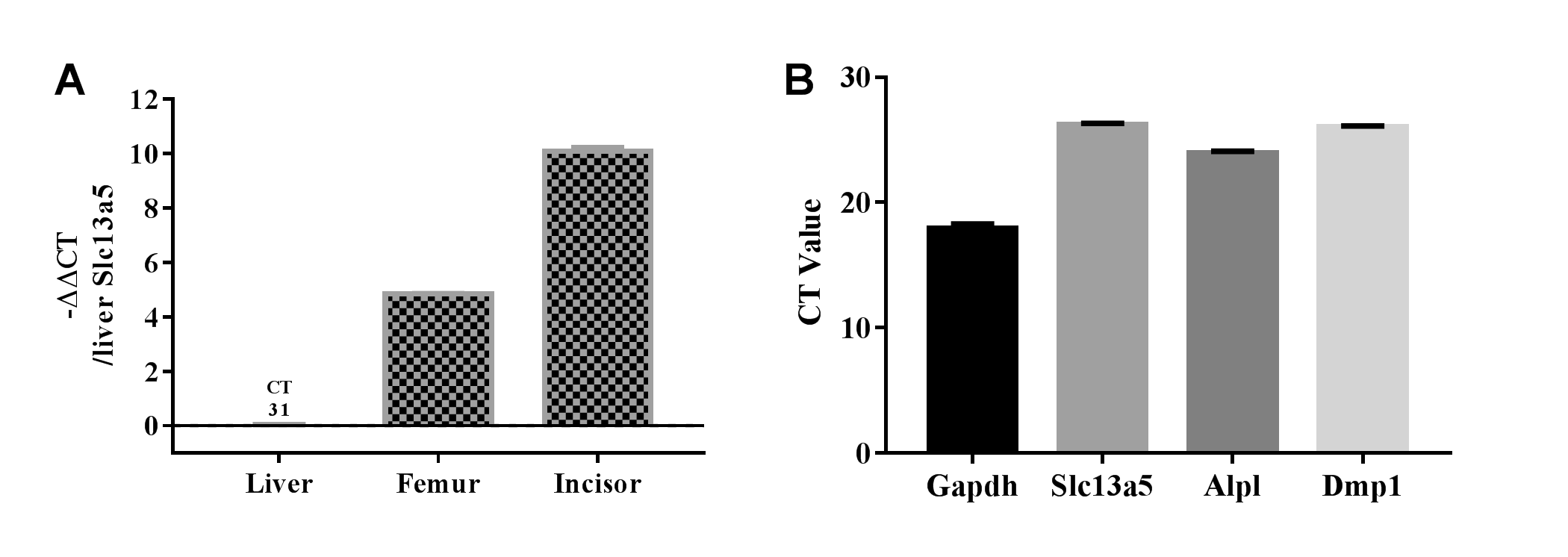

Supplement: S3 Fig — (TIF) [file pone.0175465.s003.tif]
