# Supplementary material for: Defective enamel and bone development in sodium-dependent citrate transporter (NaCT) Slc13a5 deficient mice
Source: PLoS One. 2017 Apr 13;12(4):e0175465. doi: 10.1371/journal.pone.0175465 (PMC5391028; doi:10.1371/journal.pone.0175465)
Supplement: S1 Text — (DOCX) [file pone.0175465.s004.docx]

**Supplementary Methods & Materials**

**Generation and genotyping of Slc13a5^-/-^ mice**

*Slc13a5^-/-^* mice were generated by Taconic (Köln, Germany). As shown in Supplemental Fig.1, targeting vector carrying FRT-flanked positive selection cassette (Puromycin resistance - PuroR) was generated using BAC clones from the C57BL/6J RPCIB-731 BAC library and was transfected into the TaconicArtemis C57BL/6N Tac ES cell line. Homologous recombinant clones with deletion of exon 2-12 of *Slc13a5* were isolated using positive (PuroR) and negative (Thymidine kinase - Tk) selection and then injected into BALB/c blastocysts. Chimerism was determined in chimeras (G0) by coat color contribution of ES cells to the BALB/c host (black/white). Highly chimeric mice were bred to strain C57BL/6 Flp_deleter females and germline transmission was identified by the presence of black, strain C57BL/6, offspring (G1). G1 mice continued to breed with wide type of C57BL/6 or G1 to obtain more *Slc13a5^+/-^* mice in HD Biosciences Co (HDB, Shanghai, China). The ensuing *Slc13a5^-/-^* mice without Flp gene were generated by *Slc13a5^+/-^* X *Slc13a5^+/-^*. The ensuing *Slc13a5^-/-^* , *Slc13a5^+/-^*, and *Slc13a5^+/+^* mice without Flp gene were genotyped by PCR. The primer sequences for *Slc13a5* were mixture of CAGGGAAAGGTTGGACTTGG; GAGAGGCTGTCAATGTGATATGG and ACATCTGTGCACCAGTAGACAGC. Expected fragments for *Slc13a5* wide type allele and constitutive KO allele 2 is 225 bp and 337 bp respectively. The primer sequences for *Flpe* were GGCAGAAGCACGCTTATCG and GACAAGCGTTAGTAGGCACAT, resulting 343 bp fragments. PCR control primers are GAGACTCTGGCTACTCATCC and CCTTCAGCAAGAGCTGGGGAC, resulting 585 bp fragments.

**S1 Fig. Generation and genotyping of *Slc13a5*^-/-^ mice**

1. Targeting vector carrying FRT-flanked PuroR cassette was used for homologous recombination with mouse *Slc13a5*. Positive clones (Constitutive KO allele 1) were isolated using positive (PuroR) and negative (Thymidine kinase - Tk) selection. Flp-mediated removal of the PuroR cassette resulted in Constitutive KO allele 2. Deletion of exons 2-12 resulted in loss of function of the *Slc13a5* by deleting most of the gene and by removing the 3´untranslated region (UTR). (B) PCR results of ensuing *Slc13a5^-/-^* , *Slc13a5^+/-^*, and *Slc13a5^+/+^* mice without Flp gene. 1:ddH_2_O; 2: *Slc13a5^+/-^* mice with *Flpe* gene; 3: *Slc13a5^-/-^* mice without *Flpe* gene; 4: *Slc13a5^+/-^* mice without *Flpe* gene; 5: *Slc13a5^+/+^* mice without *Flpe* gene.
