# Supplementary material for: Defective enamel and bone development in sodium-dependent citrate transporter (NaCT) Slc13a5 deficient mice
Source: PLoS One. 2017 Apr 13;12(4):e0175465. doi: 10.1371/journal.pone.0175465 (PMC5391028; doi:10.1371/journal.pone.0175465)
Supplement: S2 Text — (DOCX) [file pone.0175465.s005.docx]

**S2 Fig. Gated area in the left hemi-mandibles used to evaluate the volume of enamel in teeth.**

The image showed the gated area in left hemimandibles that was evaluated using computed tomography (white rectangle). The volumetric renderings in Fig 5 were created from a smaller area comprising the first molar instead of the larger gated area shown in Figure S2.
