# Supplementary material for: Defective enamel and bone development in sodium-dependent citrate transporter (NaCT) Slc13a5 deficient mice
Source: PLoS One. 2017 Apr 13;12(4):e0175465. doi: 10.1371/journal.pone.0175465 (PMC5391028; doi:10.1371/journal.pone.0175465)
Supplement: S3 Text — (DOCX) [file pone.0175465.s006.docx]

**S3 Fig. NaCT mRNA/*Slc13a5* expression level in teeth and bone**

qRT-PCR analysis of *Slc13a5* level in femora and incisors (A) and primary bone cells (B). (A) High levels of *Slc13a5* were present in femora and incisors. *Gapdh* was used as internal control and liver tissue was used to be positive control in which *Slc13a5* was reported to be expressed. Tissues were from male C57BL/6J mice, 7~10 week-old. Data was normalized to liver *Slc13a5* and presented as -∆∆CT. (B) *Slc13a5* was also confirmed highly expressed in primary bone cells. *Gapdh* was used as internal control. Marker genes of *Alpl and Dmp1* were tested as positive controls for osteoblasts/osteocytes. The cells were mixture from male C57BL/6J mice, 10 week-old. All primers were ordered or custom-made from Thermo Fisher Scientific (Waltham, MA, USA).
